# Use of MODIS Sensor Images Combined with Reanalysis Products to Retrieve Net Radiation in Amazonia

**DOI:** 10.3390/s16070956

**Published:** 2016-06-24

**Authors:** Gabriel de Oliveira, Nathaniel A. Brunsell, Elisabete C. Moraes, Gabriel Bertani, Thiago V. dos Santos, Yosio E. Shimabukuro, Luiz E. O. C. Aragão

**Affiliations:** 1 Remote Sensing Division, National Institute for Space Research, 1758 Astronautas Avenue, São José dos Campos, SP 12227-010, Brazil; bete@dsr.inpe.br (E.C.M.); gabrielb@dsr.inpe.br (G.B.); yosio@dsr.inpe.br (Y.E.S.); laragao@dsr.inpe.br (L.E.O.C.A.); 2 Department of Geography and Atmospheric Science, University of Kansas, 1475 Jayhawk Boulevard, Lawrence, KS 66045, USA; brunsell@ku.edu; 3 Department of Soil, Water and Climate, University of Minnesota, 1991 Upper Bufford Circle, Saint Paul, MN 55108, USA; dossa013@umn.edu; 4 College of Life and Environmental Sciences, University of Exeter, Rennes Drive, Exeter EX4 4RJ, UK

**Keywords:** Amazon region, net radiation, MODIS sensor, GLDAS data, LBA project

## Abstract

In the Amazon region, the estimation of radiation fluxes through remote sensing techniques is hindered by the lack of ground measurements required as input in the models, as well as the difficulty to obtain cloud-free images. Here, we assess an approach to estimate net radiation (Rn) and its components under all-sky conditions for the Amazon region through the Surface Energy Balance Algorithm for Land (SEBAL) model utilizing only remote sensing and reanalysis data. The study period comprised six years, between January 2001–December 2006, and images from MODIS sensor aboard the Terra satellite and GLDAS reanalysis products were utilized. The estimates were evaluated with flux tower measurements within the Large-Scale Biosphere-Atmosphere Experiment in Amazonia (LBA) project. Comparison between estimates obtained by the proposed method and observations from LBA towers showed errors between 12.5% and 16.4% and 11.3% and 15.9% for instantaneous and daily Rn, respectively. Our approach was adequate to minimize the problem related to strong cloudiness over the region and allowed to map consistently the spatial distribution of net radiation components in Amazonia. We conclude that the integration of reanalysis products and satellite data, eliminating the need for surface measurements as input model, was a useful proposition for the spatialization of the radiation fluxes in the Amazon region, which may serve as input information needed by algorithms that aim to determine evapotranspiration, the most important component of the Amazon hydrological balance.

## 1. Introduction

The Amazon region concentrates the largest rainforest in the world, which is characterized by a high amount of natural resources and for its high biodiversity of plants and animal species [1,2]. It encompasses a large area and is positioned in the tropics, where the energy exchange between the land surface and the atmosphere is at a maximum [3]. This region, which is a major source of heat and water vapor for the global atmosphere, has been the focus of attention due to the effects that large-scale deforestation can cause on local, regional, and global climate [4,5].

Radiative processes occurring on land surface are of a remarkable importance for the redistribution of moisture and heat in soil and the atmosphere [6]. Net radiation (Rn) is the difference between incoming and outgoing radiation fluxes (shortwave and longwave) at the Earth’s surface and drives, among others, the processes of photosynthesis and evapotranspiration [7]. It is a key variable for the estimation of surface energy balance and is used for a wide range of applications including hydrology, climate research, weather prediction, and agricultural meteorology [8,9]. Given the importance of physical and biological phenomena involving radiation flux exchanges, it is necessary to know these variables across both temporal and spatial scales, which allows for the improved understanding of land surface processes [10,11].

Since the early 1980s, several micrometeorological experiments have been conducted involving the collection of continuous data in Amazonia. The Amazonian Research Micrometeorological Experiment (ARME) (1983–1985) aimed to acquire information to calculate rainforest evapotranspiration rates [12]. The Amazonian Boundary Layer Experiment (ABLE) (1985–1987) was conducted in order to collect data about the structure of the atmosphere in the Amazon region [13]. The Anglo Brazilian Amazonian Climate Observational Study (ABRACOS) (1991–1995) aimed to obtain measurements of energy fluxes under the conditions of forest and pasture [14]. The 2014–2015 Green Ocean Amazon Experiment (GOAmazon) was designed to enable the study of how aerosols and surface fluxes influence cloud cycles under clean conditions, as well as how aerosol and cloud life cycles are influenced by pollutant outflow from a tropical megacity [15]. Currently, the primary source of ground data in the Amazon region refers to the Large-Scale Biosphere-Atmosphere Experiment in Amazonia (LBA) [16]. This project was designed in 1998 with the purpose to develop the knowledge about the Amazon mainly in the fields of climate physics and biogeochemistry. The LBA has flux towers on different surfaces in the Brazilian states of Rondonia (RO), Amazonas (AM), Tocantins (TO), and Para (PA). The collected data have been used to understand the present state of the Amazon environment and its response to current disturbance [17].

However, the density of ground-based measurements in Amazonia is very sparse and given the extent of this region, it is necessary to represent the spatial variability of the surface radiation fluxes at a broader scale. Furthermore, these data are often temporarily limited, which can cause difficulty in their use in certain weather and climate modeling studies. Remote sensing data can provide high spatial and temporal coverage of the land surface [18,19,20,21]. In this sense, several studies have been carried out to estimate Rn and its components combining satellite imagery with meteorological data [6,22,23,24,25,26]. Remote sensing studies often make use of information obtained from daily images, such as surface reflectance, surface temperature, emissivity, and vegetation indices. Most of these studies use sensors on board the orbital platforms of National Aeronautics and Space Administration’s (NASA) Earth Observing System (EOS) program, such as Thematic Mapper (TM-Landsat 5 satellite) and Moderate Resolution Imaging Spectroradiometer (MODIS-Terra and Aqua satellites) [20,27]. In relation to meteorological data, these studies generally make use of vapor pressure, air temperature, and incoming shortwave radiation measured from flux towers at the instant of the satellite overpass. A significant point here is that with the increase of spatial and temporal resolutions of remotely sensed images, meteorological input data to the models at reasonable temporal and spatial scales are required but irregularly available. Even though it may be possible to improve the spatial resolution of meteorological data, it is meaningful to explore different methods to retrieve the radiation fluxes at the surface. Previous studies focused on the use of reanalysis products in conjunction with satellite images to estimate Rn and its associated parameters rather than ground-based measurements [28,29,30,31]. An important source of these kind of data comes from the National Centers for Atmospheric Prediction (NCEP)/National Center for Environmental Research (NCAR) and Global Land Data Assimilation System (GLDAS) [32,33]. Although predictions from reanalyses are comprehensive in nature, the land surface products do have problems resulting primarily from the assimilation of data taken primarily from atmospheric profiles. Therefore, it is important to conduct analyzes about the biases and errors for the variables used in the models that aim to retrieve the surface radiation budget [28,34].

The literature related to the estimation of radiation fluxes in Amazonia through remote sensing techniques is quite scarce [35,36], due by the lack of ground measurements required as input in the models, as well as the difficulty obtaining sufficiently cloud-free satellite images. In this context, this paper aims to propose and assess an approach to estimate net radiation and its components under all-sky conditions for the Amazon region through the Surface Energy Balance Algorithm for Land (SEBAL) model [6] utilizing only remote sensing and reanalysis data. The study period comprises 6 years, between January 2001–December 2006, and images from MODIS sensor aboard the Terra satellite and GLDAS reanalysis products are utilized. The estimates are evaluated with flux tower measurements within the LBA project.

## 2. Materials and Methods

### 2.1. Study Area

**Figure 1:**
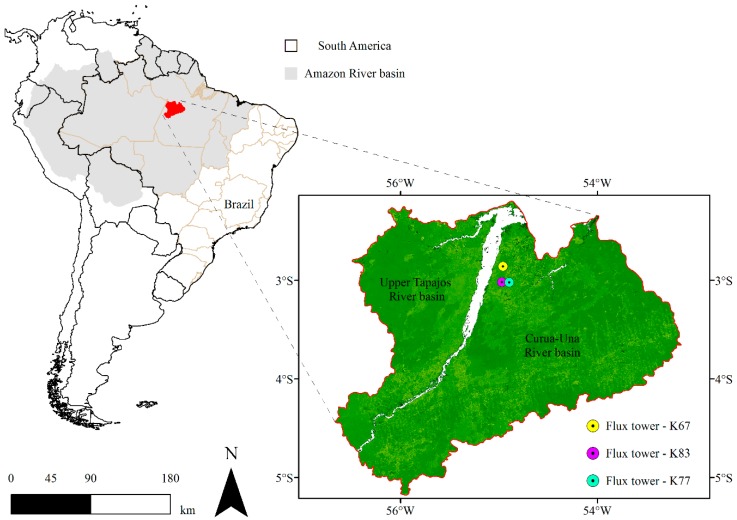
Map showing the study area and the spatial location of the flux tower sites used in this study.

The study area is located in the Upper Tapajos and Curua-Una River basins, state of Para, northern region of Brazil, between latitudes 2.19° S and 5.18° S and 56.67° W and 53.15° W (Figure 1). The study area covers 74,190 km^2^, with an average elevation of 98 m, ranging between 7 and 297 m. The predominant climate is the AmW (Köeppen classification) [37], with an annual mean precipitation of ~2000 mm and an annual mean air temperature of ~26 °C [38]. The region has well-defined dry and wet seasons. The wet season is from January to June and the dry season lasts from July through December [39]. Soils in the region are classified mainly as dystrophic yellow latosol, with high content of clay, low pH, and low content of nutrients [40,41]. The main natural vegetation type in the study area is the dense forest [42], which has been logged and replaced by agriculture in the past few decades. For this reason, it is possible to observe in the region extensive patches of different land cover types as pasture, secondary succession forest, etc.

#### Flux Tower Sites

Three eddy covariance flux towers sites of the LBA project (K67, K83 and K77) were used in this study (Figure 1). The sites are located in the municipality of Belterra, near the confluence of the Tapajos and Amazon rivers, at an average elevation of 130 m. K67 (2.86° S, 54.96° W) and K83 (3.02° S, 54.97° W) are forest experimental sites. K67 is in an undisturbed primary forest [43]. At the K83 site trees with diameter at breast height greater than 35 cm were selectively logged during three months beginning in September 2001 [44]. These sites have canopies with a mean height of ~35–40 m. The flux towers in K67 and K83 are 63 m and 64 m tall, respectively [45]. K77 (3.02° S, 54.90° W) is in a previously forested area that had been converted to pasture. The primary forest was cleared in 1990 and the field was then planted with grass. In November 2001 the site was prepared for rice cultivation. The rice was harvested in June 2002 and the field was not replanted [46]. The mean canopy height at K77 is of ~0–0.6 m and flux measurements are made on a 18 m tower [45]. Additional information about K67, K83, and K77 sites can be found in Gonçalves et al. [17], Saleska et al. [39], Rice et al. [47], and Miller et al. [48].

### 2.2. Land Cover Data

Land cover data from the TerraClass project (http://www.inpe.br/cra/ingles/project_research/terraclass.php) were used in order to analyze the dynamic of net radiation under different ecosystems in the study area. This project has been developed by National Institute for Space Research (INPE)/Brazilian Agricultural Research Corporation (EMBRAPA) and aims to map the land cover especially in areas where occurred deforestation, using satellite data. According to the TerraClass classification, there are approximately 10 different land cover types inside the Upper Tapajos and Curua-Una River basins. We selected five classes for analysis, considering they are the primary ecosystems in the study area. The selected classes were: agriculture (AG), bare soil (BS), primary tropical forest (PF), pasture (PA) and secondary succession forest (SF). Using a mapping referent to 2008 [49] and two mosaics of TM-Landsat 5 images of 2001 and 2006, we selected 12 plots of 1 km × 1 km within each land cover type. Plots were selected through the ArcGIS 9.3 software (Esri, Redlands, CA, USA, http://www.esri.com/software/arcgis) and from the visual inspection of TerraClass polygons superposed to the TM/Landsat 5 mosaics. Based on these plots, we extracted the long-term time series of Rn.

### 2.3. Observational Data

The ground data were collected at three flux towers located in K67, K83 and K77 LBA sites (described in Section 2.1). These data were acquired from the Oak Ridge National Laboratory (ORNL) (Oak Ridge, TN, USA, http://daac.ornl.gov/LBA/lba.shtml). Data were obtained, in general, between the years 2001 and 2006 but the time period varies from site to site. The temporal coverage of observational data at K67 and K83 sites are between January 2002–January 2006 and January 2001–March 2004, respectively. At the K77 site, observations span the period from January 2001 to December 2005. Flux tower measurements were used to validate the estimates of Rn and its components derived from satellite images and reanalysis products. In order to carry out this evaluation we utilized the following meteorological parameters: air temperature (T_a_), incoming (K↓) and outgoing (K↑) shortwave radiation, incoming (L↓) and outgoing (L↑) longwave radiation, and Rn. The albedo (α_s_), which is also required for validation, is not measured directly by flux towers and was calculated as the ratio of the outgoing to the incoming shortwave radiation. The LBA flux data have been processed according to a common protocol and are aggregated to an hourly time step [45]. For comparison with instantaneous estimates, we used data corresponding to 11:00 a.m. local time in the Upper Tapajos and Curua-Una River basins region (GMT-4). This period includes the Terra satellite overpass over the study area (~10:30 a.m. local time). Based on these 1-hour data, we have calculated the daily and consequently monthly averages. We chose not to apply gap-filling procedures to the datasets [50,51]. The quality control in the data followed a methodology that is applied to all LBA datasets and can be found in details in Gonçalves et al. [17] and Restrepo-Coupe et al. [45].

### 2.4. MODIS Data

Remote sensing data used in this study were derived from the MODIS sensor aboard the Terra satellite, launched in December 1999. Terra satellite crosses the study area during the daytime at approximately 10:30 a.m. local time. It should be noted that there are fewer clouds over the Amazon region in the morning in comparison with the afternoon, especially in the dry season. For this reason, it is more appropriate to use MODIS sensor images from Terra satellite instead of Aqua satellite, which passes south to north over the equator in the afternoon. The MODIS sensor has a polar orbit, a 700 km altitude and an imaging area of 2330 km [52]. The sensor has 36 spectral bands spanning 405–14385 nm wavelengths. The spatial resolution at nadir varies with spectral band: 250 m (bands 1–2) (visible), 500 m (bands 3–7) (visible/medium infrared), and 1 km (bands 8–36) (visible/thermal infrared). The geometric characteristics of this sensor ensure high accuracy in multi-spectral registration, multi-temporal registration, and absolute geolocation [53].

The MODIS datasets were obtained from the United States Geological Survey (USGS) (Sioux Falls, SD, USA, https://lpdaac.usgs.gov/data_access/data_pool). We used the following Version 5 products: MOD09Q1, MOD09A1, MOD11A2, and MOD44W. MOD09Q1 provides surface reflectance for bands 1 and 2 in an 8-day composite at 250 m resolution. MOD09A1 provides reflectance values for bands 3, 4, 5, 6 and 7 in an 8-day composite at 500 m resolution. MOD11A2 gives land surface temperature in an 8-day composite at 1 km resolution over cloud-free land areas (clouds are masked with the MODIS cloud mask data product (MOD35L2)). MOD44W corresponds to land-water mask at 250 m resolution. The images used encompassed the h12v09 tile, which covers the Upper Tapajos and Curua-Una River basins in the Amazon region, from January to December, over 6 years (2001–2006). First, MODIS images were projected to a geographic coordinate system (lat./long.) based on the WGS 84 datum, spatially resampled to 1 km using the nearest neighborhood method according the proposition of Wolfe et al. [54], and converted to the GeoTIFF format using the MODIS Reprojection Tool (MRT) (USGS, https://lpdaac.usgs.gov/tools/modis_reprojection_tool). Then, a number of steps were undertaken including clipping of the study area, multiplication by scale factors, application of the land-water mask over MOD09Q1, MOD09A1 and MOD11A2 datasets, extraction of the cloud mask from MOD11A2 product and application over MOD09Q1 and MOD09A1 datasets.

### 2.5. Reanalysis Data

Reanalysis products from GLDAS were used in this study. The GLDAS project has been developed jointly by scientists at the NASA/Goddard Space Flight Center (GSFC) and the National Oceanic and Atmospheric Administration (NOAA)/NCEP. GLDAS is a global, low resolution, offline (uncoupled to the atmosphere) terrestrial modeling system that integrates satellite and ground-based observational data in order to produce optimal fields of land surface states and fluxes in near real time [33].

The GLDAS datasets were acquired from the NASA Goddard Earth Sciences Data and Information Services Center (GESDISC, Greenbelt, MD, USA, http://disc.sci.gsfc.nasa.gov/hydrology/data-holdings). The GLDAS products used in this paper referred to the Version 1 are incoming shortwave radiation and air temperature. These products are generated with the Noah land Surface model [55] at a resolution of 0.25° and a temporal frequency of three hours (12:00 a.m., 3:00 a.m., 6:00 a.m., 9:00 a.m., 12:00 p.m., 3:00 p.m., 6:00 p.m., 9:00 p.m.). For the incoming shortwave radiation, we used the data available every three hours (8 files/day) for the entire study period. Concerning the air temperature, we only used the data corresponding to 3:00 p.m., which refers to 11:00 a.m. local time in the Upper Tapajos and Curua-Una River basins region (GMT-4). This period includes the instant of Terra satellite overpass over the study area (~10:30 a.m. local time). Preprocessing of the data included clipping of the study area, projection to a geographic coordinate system (lat./long.) based on the WGS 84 datum, spatial resampling to 1 km using the nearest neighbor method, application of the land-water mask and the cloud mask extracted from MODIS products. It should be noted that incoming shortwave radiation and air temperature products were averaged for every 8-day period according to the dates of the MODIS composite products in each year. Then, the composite images were stored in the GeoTIFF format. All procedures involving the GLDAS products were conducted aiming that they were spatially and temporally consistent with the MODIS datasets. This consistency is necessary to properly estimate the net radiation and components considering our approach.

### 2.6. Estimation of Net Radiation

The physically based SEBAL model [6] was used to calculate Rn and its components in the Upper Tapajos and Curua-Una River basins. The SEBAL model is comprised of 25 computational sub-models that computes evapotranspiration and other components of the energy balance. The components of the surface energy balance are estimated in the following sequence: Rn, ground heat (G), sensible heat (H), and latent heat (λE). In its original formulation, the model requires as input ground-based meteorological data in addition to satellite images. Therefore, to retrieve Rn SEBAL needs field measurements of air temperature and incoming shortwave radiation, and to obtain evapotranspiration estimates the model needs field measurements of wind speed [56,57].

In the following, we present the theoretical basis of SEBAL to estimate the net radiation as well as some adaptations incorporated in the model for eliminating the need of ground data as input. The SEBAL model was implemented using the ERDAS Imagine 2014 (Leica Geosystems, Heerbrugg, Switzerland, http://leica-geosystems.com/) spatial modeler function. The instantaneous Rn (W·m^−2^) (~10:30 a.m. local time) was calculated using the relationship proposed by Bastiaanssen et al. [6]:

(1) $$\mathrm{Rn}=\left(1-\alpha_{s}\right)K↓+L↓-L↑-\left(1-\varepsilon_{s}\right)L↓$$

where α_s_ is the surface albedo, K↓ (W·m^−2^) is the incoming shortwave radiation at surface, L↓ (W·m^−2^) is the incoming longwave radiation from the atmosphere, L↑ (W·m^−2^) is the outgoing longwave radiation emitted from the surface to the atmosphere, and ε_s_ is the surface emissivity.

The surface albedo (α_s_) was determined using the algorithm developed by Liang [58]:

(2) $$\alpha_{s}=0.160\rho_{1}+0.291\rho_{2}+0.243\rho_{3}+0.116\rho_{4}+0.112\rho_{5}+0.081\rho_{7}-0.0015$$

where ρ_i_ (i = 1, 2, …, 7) is the surface reflectance for bands 1 through 7 of the MODIS sensor.

To obtain the surface emissivity (ε_s_), it was necessary to calculate three vegetation indices: the normalized vegetation difference index (NDVI), the soil adjusted vegetation index (SAVI), and the leaf area index (LAI) (m^2^·m^−2^). The NDVI was calculated as the difference between near-infrared and red reflectance values normalized over the sum of the two [59]:

(3) $$\mathrm{NDVI}=\frac{\rho_{2}-\rho_{1}}{\rho_{2}+\rho_{1}}$$

The SAVI was computed as follows:

(4) $$\mathrm{SAVI}=\frac{(1+\delta )\left(\rho_{2}-\rho_{1}\right)}{\left(\delta +\rho_{2}+\rho_{1}\right)}$$

where δ is an adjustment factor used to compensate for the influence of varying soil backgrounds on the measured plant index and we are using the typically assigned value of δ = 0.5 [60].

To obtain the LAI, we used the method of Allen et al. [56].

(5) $$\mathrm{LAI}=\frac{\ln\left(\frac{0.69-\mathrm{SAVI}}{0.59}\right)}{0.91}$$

The ε_s_ was estimated from the LAI as:

(6) $$\varepsilon_{s}=0.95+0.01\mathrm{LAI}$$

Equation (6), however, is only valid for pixels with NDVI values greater than zero. For the pixels with NDVI values less than zero, we assigned ε_s_ = 0.985 [56].

In the SEBAL model, K↓ is calculated as a function of the solar constant, the solar zenith angle, the relative distance between the Earth and the Sun, and the atmospheric transmissivity at the instant of the satellite overpass [56]. In our approach, we used raster data from GLDAS related to the incoming shortwave radiation for 11:00 a.m. local time.

The incoming longwave radiation from the atmosphere (L↓) was estimated by the Stefan-Boltzmann law:

(7) $$L↓=\varepsilon_{a}\sigma {T_{a}}^{4}$$

where ε_a_ is the atmospheric emissivity, σ is the Stefan-Boltzmann constant (5.67 × 10^−8^ W·m^−2^·K^−4^), and T_a_ is the near surface air temperature (K). In the original formulation of SEBAL, the information about T_a_ is obtained from a micrometeorological tower located within the study area. In this study, aiming to eliminate the needed of observational measurements to estimate net radiation and its components, we used air temperature data obtained from GLDAS for 11:00 a.m. local time.

The atmospheric emissivity (ε_a_) was estimated through an empirical formula proposed by Bastiaanssen et al. [6]:

(8) $$\varepsilon_{a}=0.85{\left(-\ln\tau \right)}^{0.09}$$

where τ is the one way atmospheric transmissivity, computed using the equation described by Allen et al. [56]:

(9) $$\tau =0.75+2\times 10^{-5}\;z$$

where z is the elevation (m). Usually, the authors consider z the elevation in the pixel where the micrometeorological tower is located which provides ground-based data for the model. We assigned the average elevation of the study area for z, which corresponds to 98 m. We computed this average through the GTOPO30 global digital elevation model (DEM, https://lta.cr.usgs.gov/GTOPO30).

The outgoing longwave radiation emitted from the surface to the atmosphere (L↑) was estimated using the following equation:

(10) $$L↑=\varepsilon_{s}\sigma {T_{s}}^{4}$$

where T_s_ is the surface temperature (K).

After we obtained the instantaneous net radiation, we estimated the daily net radiation (Rn_24h_) (W·m^−2^) adopting a simplified formula described in Bastiaanssen et al. [57]:

(11) $$Rn_{24h}=(1-\alpha_{s})K{↓}_{24h}-110\tau$$

where K↓_24h_ is the daily incoming shortwave radiation (W·m^−2^). The SEBAL model, in its conception, uses K↓_24h_ measured from flux towers. In the present study the value of K↓_24h_ used as input in SEBAL was obtained for each day through the average of the GLDAS products available every three hours (8 files/day).

The model was run for each 8-day period from January 2001–December 2006. That is, the model was run 46 times a year totaling 276 round over the whole period. Therefore, the model estimated 276 images for each variable. The atmospheric conditions over the Amazon are often cloudy, especially in the wet season, and therefore many of these images resulted in few cloud free pixels. In order to minimize this problem and provide spatially consistent surface radiation budget maps over the study area, we generated monthly composites of each variable. To obtain the composites for each month, we used four 8-day images (corresponding to 32 days). To select which images would compose each month, we took into account the composition dates of the MODIS 8-day products in a normal (non-leap) year. Then, we calculated the average of the 8-day images for those monthly periods ignoring the cloud mask values (NoData). This means that all input cells at each location, including those with a value of NoData, were used in determining the statistic.

### 2.7. Validation

The surface radiation budget estimates and reanalysis data were compared with LBA flux tower measurements to evaluate the performance of our approach. The estimated values were extracted from one pixel (1 km × 1 km) centered on each flux tower (K67, K83 and K77) [61]. It is important to highlight that K67, K83 and K77 sites comprise the conditions of primary tropical forest, secondary succession forest and agriculture, respectively. These represent the most important terrestrial ecosystems in the Amazon. Therefore, we assume that they can represent the average environmental conditions of the study area. Four indices, including the correlation coefficient (r^2^), bias (Equation (12)), root mean square error (RMSE) (Equation (13)) and mean relative error (MRE) (Equation (14)), were used to assess the accuracy of the estimations:

(12) $$\mathrm{bias}=\frac{\left(\sum X_{\mathrm{mod}}-X_{\mathrm{obs}}\right)}{N}$$

(13) $$\mathrm{RMSE}=\sqrt{\frac{\sum (X_{\mathrm{obs}}-X_{\mathrm{mod}}{)}^{2}}{N}}$$

(14) $$\mathrm{MRE}=\frac{100}{N}\sum \left|\frac{X_{\mathrm{mod}}-X_{\mathrm{obs}}}{X_{\mathrm{obs}}}\right|$$

where X_obs_ is the flux tower observation, X_mod_ is the modelled value, and N is the number of samples. The r^2^ is used to determine the strength of the linear relationship between the estimates and measurements. Bias is a measure of how a modelled value deviates from the true value, indicating if there is under or overestimation. The RMSE is the overall error in the predictions relative to the actual measured value. The MRE is a measure of prediction accuracy, expressed as a percentage. These statistical techniques are commonly used for comparing pairs of variables and allow evaluating the error of data [62,63].

## 3. Results and Discussion

### 3.1. Spatio-Temporal Dynamics of the Components of Net Radiation over the Upper Tapajos and Curua-Una River Basins

Net radiation is the sum of all gains and losses of radiation (including shortwave and longwave) to and from the surface. In this section, we address the spatial and temporal behavior of the following components of radiation budget in the study area: incoming shortwave radiation, albedo, incoming longwave radiation, outgoing longwave radiation and net radiation.

#### 3.1.1. Incoming Shortwave Radiation

**Figure 2:**
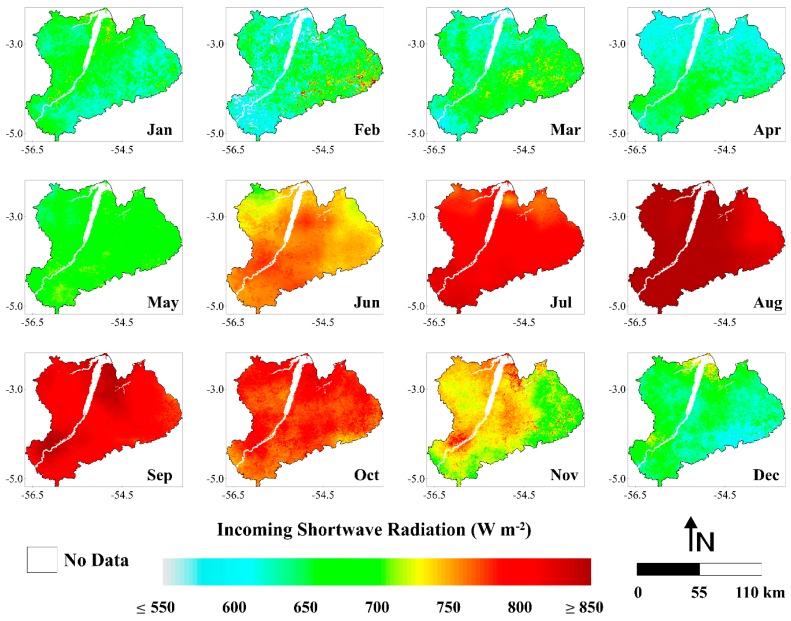
Monthly averages of incoming shortwave radiation (W·m^−2^) (months of January (Jan), February (Feb), March (Mar), April (Apr), May (May), June (Jun), July (Jul), August (Aug), September (Sep), October (Oct), November (Nov) and December (Dec)), between the years 2001 and 2006, in the Upper Tapajos and Curua-Una River basins.

The highest incidence of solar radiation throughout the year took place in the central and eastern portions of the study area (Figure 2). The images from June to November presented higher values of K↓ in comparison with the images from December to April. This pattern is attributed to the characteristics of the dry and wet seasons in the region. The dry season in the eastern part of the Amazon can vary interannually in terms of length and intensity, but typically occurs between July and December. The wet season typically occurs between January and June [39]. It is important to note that the Amazon region, located between 5° N and 10° S, receives the highest amounts of solar radiation at the top of atmosphere during the wet season but it is in the dry season that the highest amounts of solar radiation actually reach the earth’s surface [64]. According to Malhi et al. [65], the seasonal change in cloud cover is the main determinant of incoming shortwave radiation in the Amazon, with solar angle playing a secondary role. Therefore, the decreasing cloud cover during the dry season is directly associated with the increase in incoming shortwave radiation. It is important to note that greenhouse gases and smoke aerosols produced during the annual biomass-burning season in Amazonia have a substantial effect on the regional radiation budget and climate. These gases and aerosols may absorb and scatter solar radiation, leading to reductions in total solar radiation reaching the surface [66]. This reduction can cause, consequently, a decrease on the energy available at surface (Rn) to be used for the evapotranspiration process, for example. The biomass burning in the Amazon region usually occurs during the dry season (August and September), with the concentration of gases and aerosols peaking in this time of the year. Thus, depending on the quantity of fire burning events during the annual biomass-burning season, it can occur significant oscillations in the incidence of solar radiation between the driest periods of different years [67].

**Figure 3:**
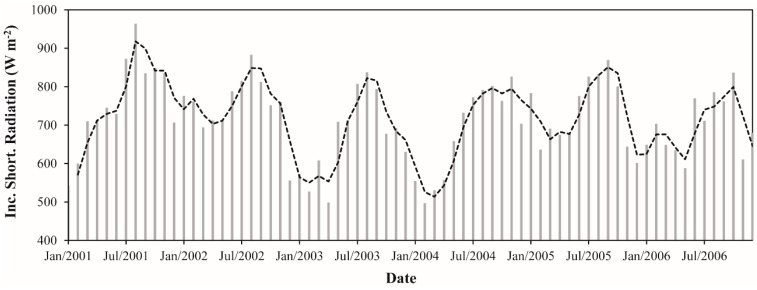
Monthly incoming shortwave radiation between January 2001 and December 2006 in the Upper Tapajos and Curua-Una River basins. The dashed black line represents the moving average of time series (period = 2).

The minimum and maximum monthly K↓ values observed were 496.7 W·m^−2^ (February 2004) and 963.9 W·m^−2^ (August 2001), denoting an absolute difference of 467.2 W·m^−2^ (Figure 3). On average for the whole study period, the monthly K↓ was 716.3 W·m^−2^. The monthly average K↓ varied between 620.7 and 849.0 W·m^−2^. February presented the lowest values of K↓ while August presented the highest ones. This can be clearly seen on the maps in Figure 2. In February, especially in the southern and northern parts, most values are ≤600 W·m^−2^, while in August, practically in the whole area, are observed values ≥850 W·m^−2^. Rocha et al. [68], in a micrometeorological study in eastern Amazonia during 2000 and 2001, observed maximum values of K↓ in September and minimum values at the beginning of the wet season. The monthly average K↓ in the wet season was 663.2 W·m^−2^ while in the dry season it was 769.4 W·m^−2^, which means an increase in K↓ during the dry season of ~16%. The annual average K↓ values ranged from 672.2 W·m^−2^ (2003) to 758.6 W·m^−2^ (2001), which corresponds to a variation of ~13%. In 2003, the year with lowest incidence of solar radiation, the monthly K↓ varied from 498.3 W·m^−2^ (May) to 837.3 W·m^−2^ (August), and in 2001 ranged between 543.0 W·m^−2^ (January) and 963.9 W·m^−2^ (August). In 2005, the Amazon basin have experienced one of the most intense drought episodes of the last 100 years [69]. In this year, the average of solar radiation reaching the surface was 734.4 W·m^−2^, which corresponds to a value ~3% than the average for the whole period (716.3 W·m^−2^). This increase occurred as expected and is most likely related to the reduction of cloudiness over Amazonia in this year especially during the wet season. During the dry season, the greenhouse gases and smoke aerosols from a large number of fire burning events that occurred in this year [70] may have contributed more than usual to absorb or scatter the solar radiation. This is probably the reason why 2005 did not present the higher value of incoming shortwave radiation considering the six years analyzed.

#### 3.1.2. Albedo

**Figure 4:**
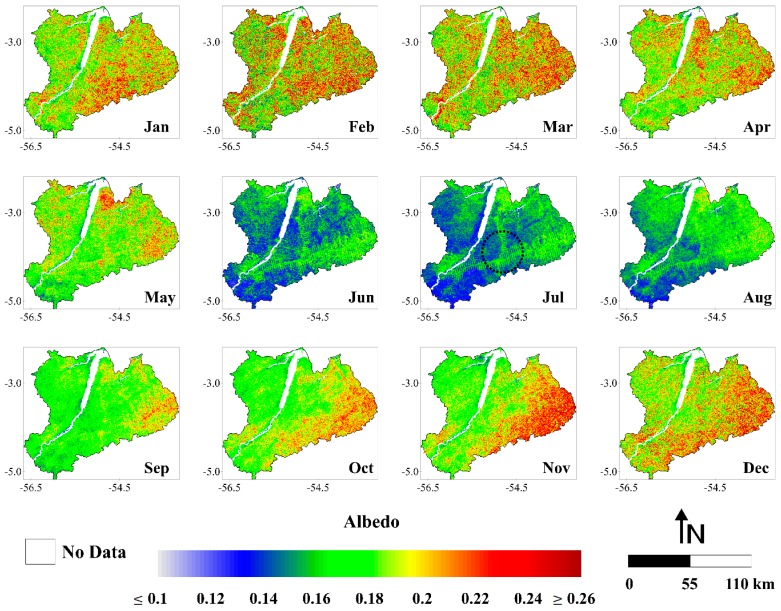
Monthly averages of albedo (months of January (Jan), February (Feb), March (Mar), April (Apr), May (May), June (Jun), July (Jul), August (Aug), September (Sep), October (Oct), November (Nov) and December (Dec)), between the years 2001 and 2006, in the Upper Tapajos and Curua-Una River basins.

The images with the highest values of albedo corresponded to the months from October to May (Figure 4). These images presented values generally ≥0.18 while the maps of June, July, August and September showed values typically ≤0.16. As can be noted, there is a decrease in albedo in the early dry season. Local-scale studies developed in Amazonia have been shown the highest albedo values generally occurring at the same time as the driest soil moisture conditions (dry season) [14,71]. Although the results here are different, we justify this behavior by the high increase of solar radiation reaching the earth surface in this region at this time of the year. That is, the decrease in surface moisture, which can causes a lower absorption of solar radiation (and consequently a higher albedo), is cancelled by a large increase in incoming shortwave radiation, resulting in a reduction of albedo. It is interesting to note that in most images, the highest values of albedo are located in the eastern part of the study area, more precisely in the Curua-Una River basin. It can be clearly seen between June and December. This area is situated within the so-called arc of deforestation, which is under higher pressure from human settlement and where the deforestation actually occurs [72]. We can also observe in the black dashed circle over the image of July the spatial pattern of deforestation in the Amazon, known as the fish spine [73]. In this regard, it is possible to verify the difference of albedo values between logged and forested areas, with the later presenting lower values. Because they are darker, forests absorb more solar radiation than short vegetation, having thus a smaller albedo [74,75,76,77].

**Figure 5:**
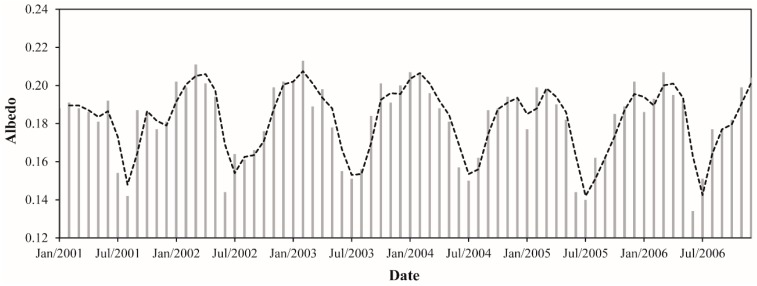
Monthly albedo between January 2001 and December 2006 in the Upper Tapajos and Curua-Una River basins. The dashed black line represents the moving average of time series (period = 2).

The monthly albedo values ranged from 0.134 to 0.213, a relative difference of ~59%. These values were observed in June 2006 and February 2003, respectively (Figure 5). Considering the average between 2001 and 2006, the monthly albedo corresponded to 0.182. Regarding the monthly average, July and February were the months with lowest (0.152) and highest (0.20) albedo values, respectively. Also, the monthly average albedo is ~5% lower in the dry season compared with the wet season. For the dry season, the monthly average albedo was 0.177 while in the wet season was 0.187. The annual average albedo values varied between 0.178 (2005) and 0.185 (2002 and 2003), constituting an absolute difference of 0.007 (~4%). This result evidence a small interannual variation of albedo in the eastern flank of Amazonia.

#### 3.1.3. Incoming Longwave Radiation

**Figure 6:**
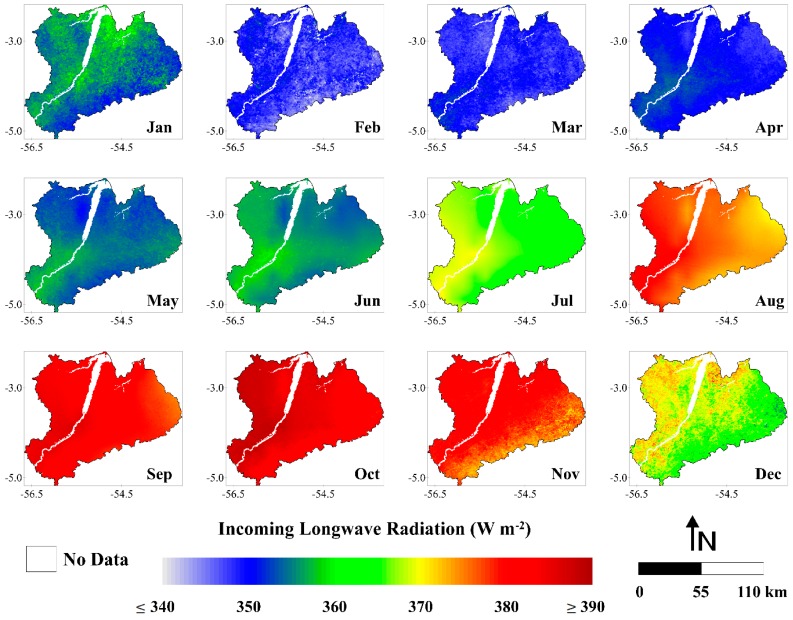
Monthly averages of incoming longwave radiation (W·m^−2^) (months of January (Jan), February (Feb), March (Mar), April (Apr), May (May), June (Jun), July (Jul), August (Aug), September (Sep), October (Oct), November (Nov) and December (Dec)), between the years 2001 and 2006, in the Upper Tapajos and Curua-Una River basins.

In Figure 6, we can note that the highest amounts of L↓ reaching the surface during the year are generally located in the western side of the study area (Upper Tapajos River basin). This is because this basin contains a larger forest area than the Curua-Una River basin. Several studies show that L↓ in forest is higher than the pasture or agricultural areas [75,78]. The lowest values of L↓ were observed between January and June while the hightest ones were observed from July to December. During the wet season (January to June) the values were generally ≤360 W·m^−2^. For the dry season (July to December) observed values were mostly ≥370 W·m^−2^. As we described in Section 2.5, L↓ was estimated by the Stefan-Boltzmann law, therefore being greatly influenced by the air temperature. According Rocha et al. [68], the dry season air temperature is ~1–3 °C higher than the wet season for the region. Analyzing the air temperature data from GLDAS we found that air temperature in the study area was ~5 °C higher in the dry season in comparison with the wet season. Therefore, the increase of L↓ in the dry season occurred as expected.

**Figure 7:**
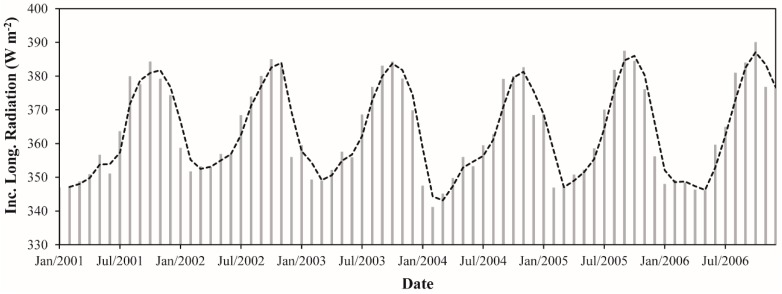
Monthly incoming longwave radiation between January 2001 and December 2006 in the Upper Tapajos and Curua-Una River basins. The dashed black line represents the moving average of time series (period = 2).

The monthly L↓ ranged from 341.2 W·m^−2^ (February 2004) to 390.1 W·m^−2^ (October 2006), representing an absolute difference of 48.9 W·m^−2^ (~14%) (Figure 7). The average monthly L↓ for the whole period was 363.9 W·m^−2^. The lowest and highest values of monthly average L↓ happened in the beginning of the dry season (February) and late wet season (October) and corresponded to 347.6 and 384.7 W·m^−2^, respectively. The monthly average L↓ in in the wet season was 352.0 W·m^−2^ while in the dry season was 375.8 W·m^−2^. This result shows an increase in L↓ during the dry season of ~7%. The annual average L↓ values varied between 360.5 and 365.5 W·m^−2^, revealing a relative difference of only 1%. The years of 2004 and 2003 presented the lowest and highest values of L↓, respectively.

#### 3.1.4. Outgoing Longwave Radiation

**Figure 8:**
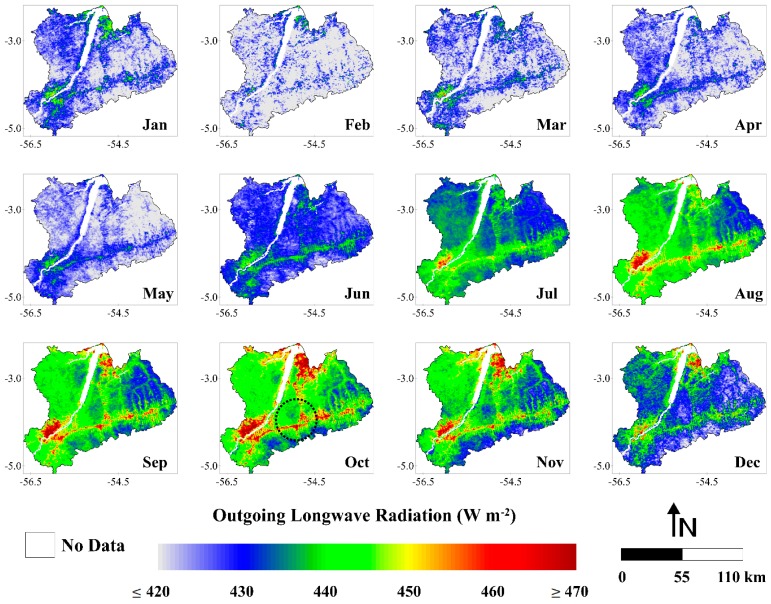
Monthly averages of outgoing longwave radiation (W·m^−2^) (months of January (Jan), February (Feb), March (Mar), April (Apr), May (May), June (Jun), July (Jul), August (Aug), September (Sep), October (Oct), November (Nov) and December (Dec)), between the years 2001 and 2006, in the Upper Tapajos and Curua-Una River basins.

The spatial pattern of L↑ reveals that the eastern portion of the study area has the highest emission of longwave radiation from the surface (Figure 8). As we discussed in Section 3.1.2, this region is strongly affected by deforestation. The conversion from forest to pasture or agricultural areas increases the surface temperature and alters the emissivity, resulting in an increase in L↑ [78,79]. The fish spine pattern is clearly observed in the images from July to December (black dashed circle over the image of October). The images with the highest values of L↑ corresponded to the months from July to December (dry season), following the pattern observed for L↓ in Section 3.1.3. These images presented values generally ≥450 W·m^−2^ while the images of wet season (January to June) showed values generally ≤430 W·m^−2^. The estimation of L↑, similarly of L↓, was obtained through Stefan-Boltzmann law (described in Section 2.5). The primary difference in the estimation relies on the use of surface temperature instead of the atmospheric temperature. The MODIS surface temperature in the study area increased in the dry season about 2.4 °C relative to the wet season, justifying the increment in L↑ during this time of the year.

**Figure 9:**
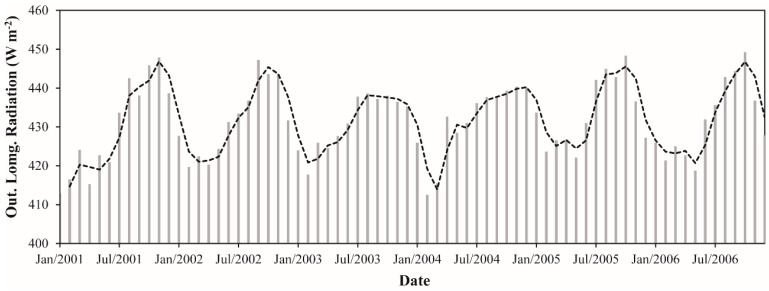
Monthly outgoing longwave radiation between January 2001 and December 2006 in the Upper Tapajos and Curua-Una River basins. The dashed black line represents the moving average of time series (period = 2).

The monthly L↑ ranged between 412.5 and 449.2 W·m^−2^, representing an absolute difference of 36.7 W·m^−2^ (~9%) (Figure 9). These values were observed in February 2004 and October 2006, respectively. It is important to note that, as expected, the minimum and maximum values of L↓ were also found in February 2004 and October 2006, respectively. Considering the average during whole study period, we observed a monthly L↑ of 431.7 W·m^−2^. In terms of the monthly average, February and October showed the lowest (418.6 W·m^−2^) and highest values (444.0 W·m^−2^), respectively. A monthly average L↑ ~4% higher in the dry season in comparison with the wet season was also observed. For the wet season, the monthly average L↑ was 424.0 W·m^−2^ while in the dry season was 439.3 W·m^−2^. The annual average L↑ values varied between 429.9 W·m^−2^ (2001) and 433.8 W·m^−2^ (2005). It constitutes a variation of ~1%, the same verified for L↓ in the Section 3.1.3. These results demonstrate a very small interannual fluctuation in the longwave radiation components over the Upper Tapajos and Curua-Una River basins.

#### 3.1.5. Net Radiation

**Figure 10:**
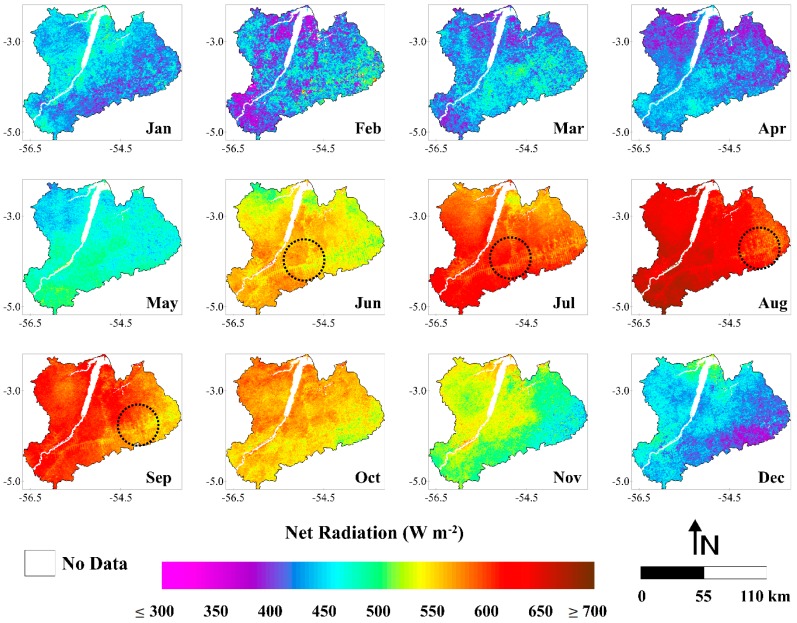
Monthly averages of net radiation (W·m^−2^) (months of January (Jan), February (Feb), March (Mar), April (Apr), May (May), June (Jun), July (Jul), August (Aug), September (Sep), October (Oct), November (Nov) and December (Dec)), between the years 2001 and 2006, in the Upper Tapajos and Curua-Una River basins.

The highest values of Rn are shown to be situated in the western part of the study area (Figure 10). As discussed before, this area is better preserved in terms of deforestation. Rn over forest areas is higher than in pasture areas, due to differences in the outgoing solar radiation (albedo) and in the longwave radiation balance [75,80]. In the images of June, July, August and September is possible to verify the fish spine pattern (black dashed circles), also clearly observed in the albedo images (Section 3.1.2) and L↑ (Section 3.1.4). This data shows that logged areas, mostly of which were converted posteriorly to pasture, present higher albedo and L↑ and lower Rn, compared to forested areas. This result is in agreement with various observational studies in the Amazon [3,65,81,82].

Rn values are higher from June to November compared to the December to April period. This is the same pattern observed for the incoming shortwave radiation (described in Section 3.1.1). It occurs due the strong and linear correlation between solar radiation and net radiation in Amazonia, noticed by several previous studies [12,75]. As discussed in Section 3.1.1, the dry season has the highest amounts of solar radiation reaching the surface in the Amazon region because of the reduction in cloud cover. This is predicted even considering the large presence of smoke aerosols from fire burning events during this time of the year, which may cause reductions of total solar irradiance [64,65,66,67]. Therefore, with the reduction in cloud cover and consequent increase in K↓, Rn increases during the driest period of the year as expected, demonstrating the effectiveness of our methodology to capture the seasonal variation of Rn in the study area.

**Figure 11:**
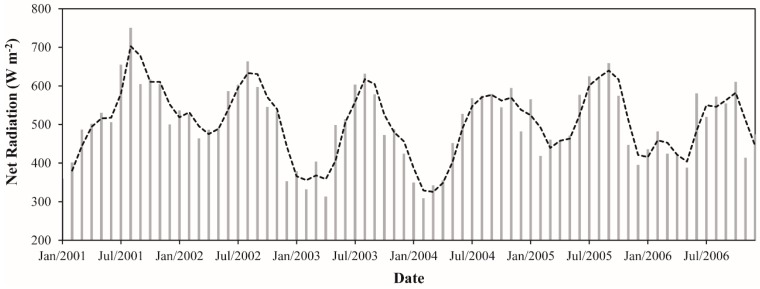
Monthly net radiation between January 2001 and December 2006 in the Upper Tapajos and Curua-Una River basins. The dashed black line represents the moving average of time series (period = 2).

The monthly Rn varied between 308.8 (February 2004) and 750.7 W·m^−2^ (August 2001). It denotes an absolute difference of 441.9 W·m^−2^ (~143%) (Figure 11). The monthly averaged Rn over the period was 505.5 W·m^−2^. The monthly average ranged from 411.7 to 635.6 W·m^−2^, with February presenting the lowest value and August presenting the highest one. We can note that in February most values are ≤450 W·m^−2^, while in August the values are generally ≥600 W·m^−2^ (Figure 10). Hutyra et al. [43] in a micrometeorological study developed in this region also found minimum and maximum values of Rn at the beginning of wet and dry season, respectively. The monthly average Rn in the wet and dry season was 453.8 and 556.7 W·m^−2^, respectively, showing that Rn increased ~23% in the dry season. This result is in agreement with the study of Costa et al. [83], which observed for the same region an increase of about 18% in Rn during the dry season using surface data. The annual average Rn values varied from 470.0 to 543.2 W·m^−2^, which corresponds to a variation of ~16%. This result shows a large interannual variation of Rn in the study area. It should be noted that, between all the parameters analyzed, Rn presented the highest interannual variation (K↓ = 13%, albedo = ~4%, L↓ and L↑ = ~1%). The years with the lowest and highest amounts of energy available at the surface were 2003 and 2001, respectively. These years coincide exactly with the years with smallest and largest amounts of solar radiation reaching the surface in the region, as discussed in Section 3.1.1. In 2005, when a severe drought occurred over the Amazon region [69,70], we observed an average Rn value ~3% higher than the annual average for the whole period. This increment is related to the increase of K↓ observed in this year, which was also of about ~3% (Section 3.1.1). As discussed previously, it probably occurred due to the reduction of cloud cover especially during the wet season in this year. We highlight that 2005 was not the year with highest value of Rn in the temporal series probably because of the large amounts of greenhouse gases and aerosols from fire burning events were released to the atmosphere in the dry season of this year, contributing to either the absorption or scattering of the solar radiation.

### 3.2. Temporal Dynamics of Net Radiation for Different Land Cover Types

**Figure 12:**
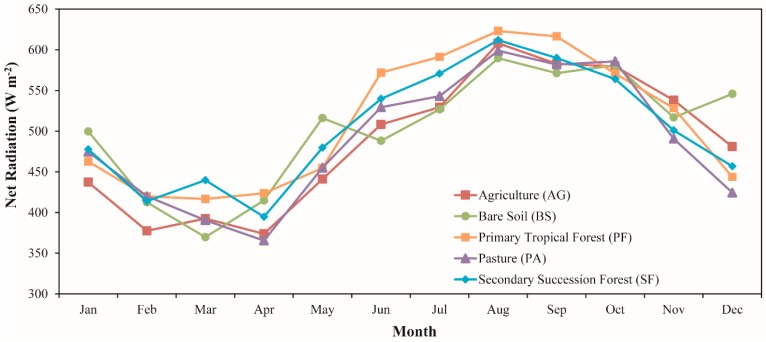
Seasonal patterns of net radiation under agriculture (AG), bare soil (BS), primary tropical forest (PF), pasture and secondary succession forest (SF). The monthly average was calculated based on the period between 2001 and 2006.

Figure 12 shows the trajectories of instantaneous net radiation for five different land cover types during the period from 2001 to 2006. The land cover types analyzed corresponded to agriculture (AG), bare soil (BS), primary tropical forest (PF), pasture (PA) and secondary succession forest (SF). Overall, most of the land cover types had a similar monthly variation, with a maximum in the dry season and a minimum in the wet season. It follows the pattern observed for the whole study area, as discussed in Section 3.1.5. Considering the average for the entire study period, the highest ecosystem Rn value was for BS (534.0 W·m^−2^), while AG presented the lowest one (495.6 W·m^−2^). We observed that the highest and lowest Rn values found for BS and AG are related, respectively, to the lower and higher albedo observed in these ecosystems. The monthly Rn varied from 369.7 (March 2005) to 657.5 W·m^−2^ (October 2001) and from 211.2 (January 2004) to 722.9 W·m^−2^ (October 2001) for BS and AG, respectively. The PA presented a temporal average Rn of 496.7 W·m^−2^, very similar to that found in AG. Wright et al. [84] and Galvao and Fish [85] observed for pasture sites in the Brazilian states of Amazonas and Rondonia, respectively, at 11:00 a.m., values about 500 W·m^−2^, which are close to those we found in our study. The monthly average Rn in the wet and dry season was 444.8 and 540.7 W·m^−2^, respectively.

In the PF, we observed an average value of 511.6 W·m^−2^. Micrometeorological studies developed by Von Randow et al. [71] and Aguiar [86] observed values of 450 and 530 W·m^−2^ in an LBA forest site in the southwestern part of Amazonia (average for 11:00 a.m. local time). Rn varied between 459.4 and 562.2 W·m^−2^ in the wet and dry season, respectively, meaning an increase during the dry season of about 22%. It is important to notice that Rn in PF was approximately 3% higher than the PA and AG, illustrating that deforestation influences the availability of energy at the surface, leading, among others, to consequences for evapotranspiration [71,87,88,89]. With respect to SF, an average value for the study period of 507.5 W·m^−2^ was observed ranging between 293.6 W·m^−2^ (March 2004) and 742.3 W·m^−2^ (August 2001). The Rn during wet and dry season was about 460.8 and 549.0 W·m^−2^, respectively, showing a variation of ~19%. It is not possible to guarantee what is exactly the age of the secondary forest plots analyzed, but it is interesting to note that the Rn is only ~1% lower to that observed for the primary forest. It shows that with time, the secondary forest can recover its original state in terms of radiation fluxes, consequently reducing the impact of the deforestation on the climatology of Amazon region.

### 3.3. Validation of Reanalysis Data

The MRE in the incoming shortwave radiation was approximately 10%–11% of the field measurements (Table 1). The bias varied from 45.7 to 70.5 W·m^−2^, while the RMSE ranged from 72.7 to 86.5 W·m^−2^. Baik and Choi [62], comparing K↓ obtained from satellite images and ground data, observed biases and RMSE varying between −21.6 to −112.6 W·m^−2^ and 107.1 to 202.4 W·m^−2^, respectively. The average r^2^ was 0.82, oscillating between 0.78 and 0.86 in K77 and K83 sites respectively. In a study developed by Shi and Liang [31] to analyze K↓ acquired from four different reanalysis datasets, it was found r^2^ values varying between 0.42 and 0.47. In respect to daily incoming shortwave radiation, considering the two sites, the MRE was about 17%. The bias and RMSE values were of the order of 25.4 to 36.5 W·m^−2^, and 32.2 to 41.2 W·m^−2^, respectively. In a daily basis, Hou et al. [9], observed biases and RMSE ranging from 21 to 25 W·m^−2^ and 27 to 28 W·m^−2^, respectively. The average r^2^ value verified in our study was 0.76 while Hou et al. [9] observed a value of 0.98. In general, considering the incoming shortwave radiation (both instantaneous and daily) it is important to note that the values tend to be overestimated in comparison of ground data. Also, it is verified that the results were more accurate for the pasture (K77) than the forest site (K83).

The air temperature presented the more accurate results of all reanalysis data analyzed in this study. In average for the three sites, the MRE was ~5%, varying between 3.7% (K77) and 6.6% (K67). For K83 was observed an MRE of 6.1%. In the same manner as verified for K↓ and K↓_24h_, were found a higher agreement between GLDAS and LBA data under the condition of pasture than forest. The average bias and RMSE corresponded to 1.2 and 2 °C, respectively, and r^2^ was of 0.80. As we can note, air temperature values from GLDAS were overestimated. Bisht et al. [8], using MODIS images to estimate air temperature, observed a bias, RMSE and r^2^ of −2.1 °C, 5 °C and 0.62 °C, respectively. Based on the results obtained, we highlight that our hypothesis to use reanalysis data as input in SEBAL model is valid and can serve as alternative to the use of ground data in regions where there is a lack of such information.

### 3.4. Validation of Model Estimates

The results presented here encompass all of the estimates using MODIS images between 2001 and 2006, except when was NoData value (cloud mask) in the satellite dataset or error in ground observations. The albedo presented the highest MRE values between all the components of net radiation (Table 2). In average for the K83 and K77 sites, the MRE corresponded to ~32%. The MRE in K83 was 43.4% while in K77 was 20.1%. There was an overestimation of 0.044 and 0.028 for the forest and pasture sites, respectively. In average, the RMSE was of 0.047 and r^2^ was of 0.16. These results reveal a certain ineffectiveness of Liang’s approach (described in Section 2.5) to retrieve the albedo through MOD09 products in the Amazon region. In a study validating the MCD43B3 product with observational data, Hou et al. [9] verified that albedo values were underestimated with a bias, RMSE and r^2^, in average, of −0.024, 0.029 and 0.32, respectively. Using different remote sensing datasets in Tibetan Plateau, Shi and Liang [31] observed RMSE and r^2^ values of the order of 0.06 and 0.07, and 0.04 and 0.10, respectively.

Concerning the longwave components of the surface radiation budget, we found an MRE, in average, of ~16% for the portion that is emitted from the atmosphere to the surface and ~9% for portion that is emited from the surface to the atmosphere. Bisht and Bras [90] analyzing the accuracy of L↓ and L↑ estimated also from MODIS images found errors between ~4% and ~8% and ~9% and ~12%, respectively. For both components were observed an underestimation, in average, of −68.6 and −41.5 W·m^−2^, for incoming and outgoing longwave radiation, respectively. The RMSE ranged from 66.6 to 73.9 W·m^−2^ for the incoming portion and from 37.1 to 47.9 W·m^−2^ for the outgoing portion. The correlation coefficient, in average, was about 0.06 and 0.47 for L↓ and L↑, respectively. Oliveira and Moraes [36] observed biases between −55.8 and 13.4 W·m^−2^ (−21.2 W·m^−2^ in average), and −41.8 and −15.3 W·m^−2^ (−28.6 W·m^−2^ in average) for incoming and outgoing longwave radiation, respectively. In regard to RMSE, Bisht and Bras [90] verified values for L↓ between 28.2 and 37.6 W·m^−2^, and for L↑ ranging from 17.1 to 35.3 W·m^−2^. The r^2^ obtained by Shi and Liang [31] were about 0.12 and 0.02 for L↓ and L↑, respectively. We can observe, in general, that the results for the longwave components were better for the outgoing portion. This is in accordance other studies which point out that the longwave radiation emitted by atmosphere into the surface is one of the components of radiation budget more difficult to retrieve [79,91]. As described in Section 2.5, the SEBAL model uses the Stefan-Boltzmann law to obtain L↓, assuming that the atmospheric emissivity can be computed through an empirical formula based on atmospheric transmissivity, which is a function primarily of the altitude of the study area. We consider that this is a very simple assumption, and according to various authors [92,93] the accurate estimation of L↓ relies on information about greenhouse gas concentrations and profiles of moisture and air temperature. However, these kind of data are extremely costly to obtain and compile.

**Figure 13:**
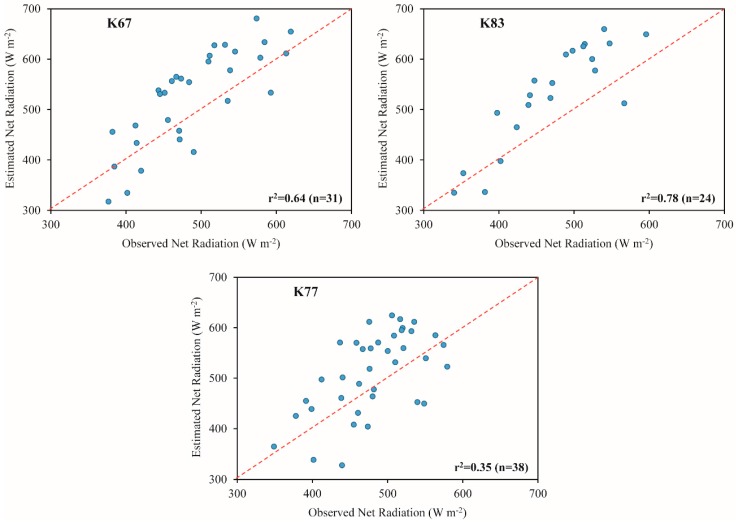
Comparison of observed and estimated net radiation at K67, K83 and K77 LBA sites. The number of correlative measurements is represented by “n”.

**Figure 14:**
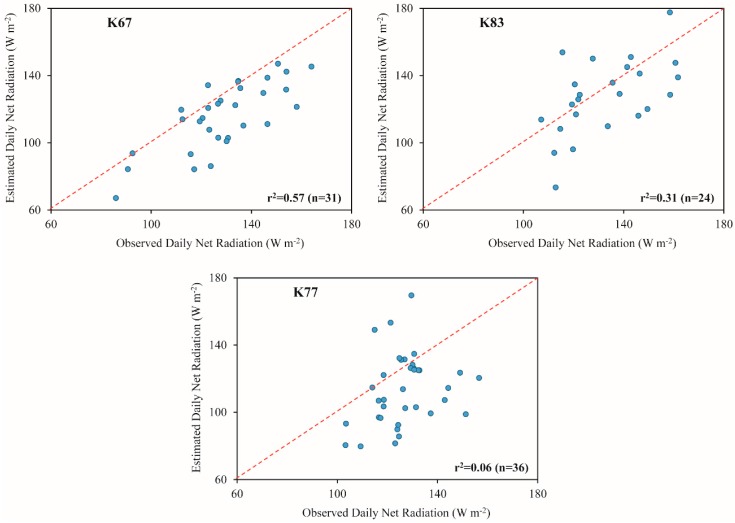
Comparison of observed and estimated daily net radiation at K67, K83 and K77 LBA sites. The number of correlative measurements is represented by “n”.

The MRE in instantaneous and daily net radiation were, in average for the three sites, of the order of 14% and 13%, respectively. Other studies also using orbital images presented errors in Rn and Rn_24h_ ranging between ~13% and ~24% [90] and ~12% and ~14% [9], respectively. The Rn tended to be overestimated, with biases varying between 30.5 and 55.1 W·m^−2^, while the Rn_24h_ tended to be underestimated, with biases ranging from −5.4 and −13.0 W·m^−2^. In average, the biases for Rn and Rn_24h_ corresponded to 40.8 and −10.5 W·m^−2^. Bisht et al. [8] verified biases of 59 W·m^−2^ (Rn) and 50 W·m^−2^ (Rn_24h_). The RMSE was, in average, 76.2 and 21.1 W·m^−2^ for Rn and Rn_24h_, respectively. The RMSE values were very close to those found by the studies of Bisht et al. [8] and Shi and Liang [31]. The first one obtained a RMSE for Rn of ~74 W·m^−2^ while the second verified a RMSE for Rn_24h_ of ~24 W·m^−2^. The r^2^ varied from 0.35 to 0.78 for Rn and from 0.06 to 0.57 for Rn_24h_ (as shown in Figure 13 and Figure 14). For both Rn and Rn_24h_, the lowest correlation values were verified at K77 site. In average, the r^2^ was about 0.60 and 0.31 for Rn and Rn_24h_, respectively. Comparing with other studies also estimating this parameters through MODIS images, we observed r^2^ values of the order of 0.78 (Rn) [94] and 0.57 (Rn_24h_) [30].

**Figure 15:**
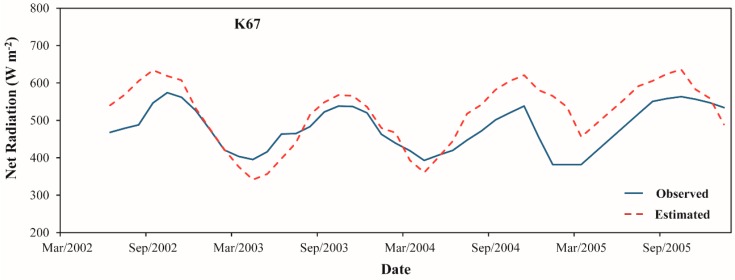

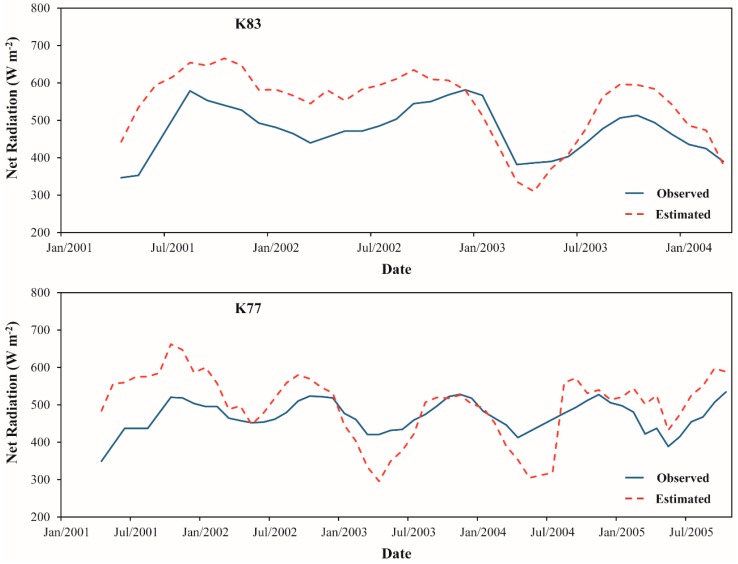
Moving average of the monthly cycle of net radiation estimated from SEBAL model and observed in K67, K83 and K77 LBA sites. The period of moving average is equal to 4.

**Figure 16:**
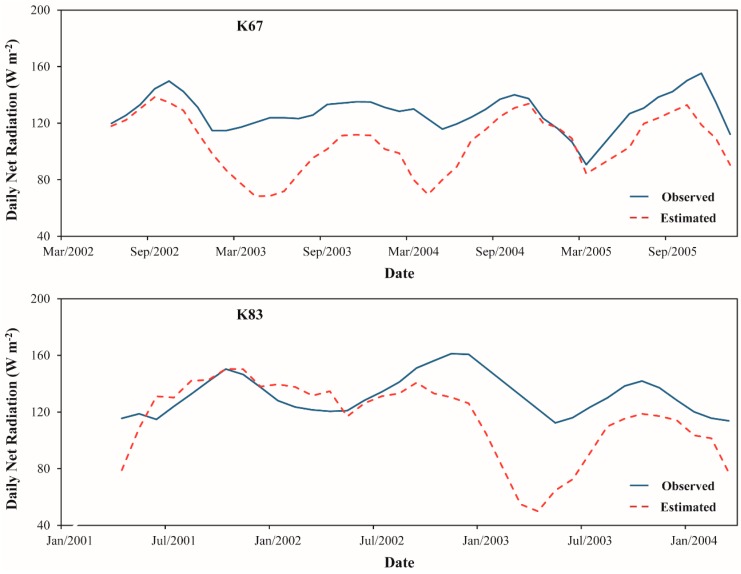

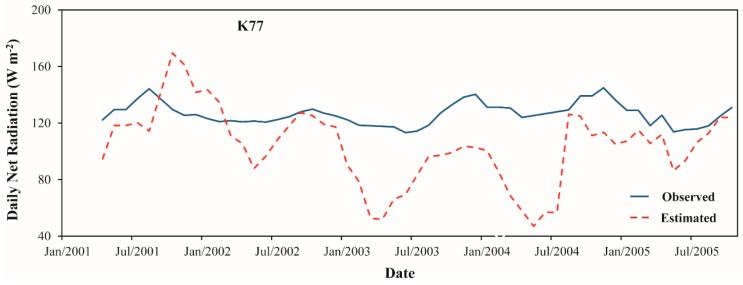
Moving average of the monthly cycle of daily net radiation estimated from SEBAL model and observed in K67, K83 and K77 LBA sites. The period of moving average is equal to 4.

Figure 15 and Figure 16 compare the seasonal variations in Rn and Rn_24h_ estimated from SEBAL model against the ground measurements over K67, K83 and K77 LBA sites. Although there are some differences of magnitude, which were explained before in terms of biases and RMSE, we can notice, in general, that our methodology was adequate to obtain the correct seasonal pattern of Rn and Rn_24h_ in the Amazon region. For both Rn and Rn_24h_, we highlight that the major discrepancy between the observed and estimated seasonal signal was found in K77 site. For the three study sites, it is clearly verified the trend of increase in the energy available at the surface to be converted to ground, sensible and latent heat fluxes during the dry season in comparison with wet season. As discussed in Section 3.1, this fact is strongly related to the seasonal variation of incoming solar radiation over the region [64,65].

## 4. Conclusions

In the present study, we developed an approach to estimate net radiation and its components under all-sky conditions at a regional scale in Amazonia using only reanalysis and remotely sensed data. Our approach aimed to eliminate the need for ground observations as model input, given the difficult to obtain such information in the Amazon region. The proposition of generating monthly composites of each variable was adequate to minimize the problem related to strong cloudiness over the region and allowed to map consistently the spatial distribution of net radiation components in the eastern part of Amazonia. In general, the spatial variation of Rn was related to the larger presence of forested areas in Upper Tapajos River basin (western flank) in comparison with Curua-Una River basin (eastern flank). Temporally, Rn have shown a strongly seasonal pattern, varying according with dry and wet seasons in the region. There is a pattern of declining of cloud cover during the dry season in Amazonia and it is directly associated with the increase in incoming shortwave radiation. We highlight that it occurred as expected due to the fact of incoming solar radiation is the main controller of Rn in the region.

Regarding the dynamic of Rn under different land cover types, the results showed, overall, that the primary forest has in long-term average a higher Rn value in comparison with pasture and agriculture, which is consistent with several meteorological studies performed in the region. It is important to note that the secondary forest presented an Rn value very close to the primary forest, showing that the preservation of these areas could serve to reduce the impact of the deforestation in the climatology of Amazonia. In addition, these results show that the use of MODIS and GLDAS data allowed discriminating, in terms of magnitude and temporal behavior, different land cover types in the region.

The reanalysis products presented errors from 3.9% to 19.5% in relation to the ground measurements, suggesting that these data were an adequate alternative as model input to estimate the surface radiation budget in the region. Comparison between estimates obtained by the proposed method and observations from the LBA towers showed errors between 12.5% and 16.4% and 11.3% and 15.9% for instantaneous and daily Rn, respectively. In order to enhance the accuracy of the present methodology, future effort should focus on reducing the errors in the estimation of albedo and L↓.

We conclude that the integration of reanalysis products and satellite data, eliminating the need for surface measurements as input model, was a useful proposition for the spatialization of the radiation fluxes in the Amazon region, which may serve as input information needed by algorithms that aim to determine evapotranspiration, the most important component of the Amazon hydrological balance.

## Figures and Tables

**Table 1:** Mean observed and estimated values, bias, root mean square error (RMSE), correlation coefficient (r^2^) (*p* < 0.05) and mean relative error (MRE) (%) for incoming shortwave radiation, daily incoming shortwave radiation and air temperature.

		K67	K83	K77
Incoming Shortwave Radiation (K↓) (W·m^−2^)	Mean Obs.	--	637.1	667.6
	Mean Est.	--	707.6	713.3
	bias	--	70.5	45.7
	RMSE	--	86.5	72.7
	r^2^	--	0.86	0.78
	MRE (%)	--	11.1	9.9
Daily Incoming Shortwave Radiation (K↓_24h_) (W·m^−2^)	Mean Obs.	--	189.5	200.9
	Mean Est.	--	226.0	226.3
	bias	--	36.5	25.4
	RMSE	--	41.2	32.2
	r^2^	--	0.81	0.71
	MRE (%)	--	19.5	14.0
Air Temperature (T_a_) (°C)	Mean Obs.	27.2	27.4	28.9
	Mean Est.	29.0	29.0	29.1
	bias	1.8	1.6	0.2
	RMSE	2.3	2.3	1.4
	r^2^	0.84	0.77	0.78
	MRE (%)	6.6	6.1	3.7

**Table 2:** Mean observed and estimated values, bias, root mean square error (RMSE), correlation coefficient (r^2^) (*p* < 0.05) and mean relative error (MRE) (%) for albedo, incoming and outgoing longwave radiation, net radiation and daily net radiation.

		K67	K83	K77
Albedo	Mean Obs.	--	0.110	0.169
	Mean Est.	--	0.153	0.197
	bias	--	0.044	0.028
	RMSE	--	0.052	0.041
	r^2^	--	0.18	0.14
	MRE (%)	--	43.4	20.1
Incoming Longwave Radiation (L↓) (W·m^−2^)	Mean Obs.	--	436.8	441.0
	Mean Est.	--	371.5	369.2
	bias	--	-65.3	-71.8
	RMSE	--	66.6	73.9
	r^2^	--	0.06	0.05
	MRE (%)	--	14.9	16.2
Outgoing Longwave Radiation (L↑) (W·m^−2^)	Mean Obs.	--	468.7	486.9
	Mean Est.	--	432.3	440.4
	bias	--	-36.4	-46.5
	RMSE	--	37.1	47.9
	r^2^	--	0.34	0.59
	MRE (%)	--	7.8	9.5
Net Radiation (Rn) (W·m^−2^)	Mean Obs.	489.0	466.6	480.7
	Mean Est.	525.6	521.6	511.2
	bias	36.6	55.1	30.5
	RMSE	68.2	88.8	71.6
	r^2^	0.64	0.78	0.35
	MRE (%)	12.5	16.4	13.1
Daily Net Radiation (Rn_24h_) (W·m^−2^)	Mean Obs.	128.9	132.9	126.6
	Mean Est.	115.9	127.4	113.6
	bias	-13.0	-5.4	-13.0
	RMSE	18.7	19.7	24.8
	r^2^	0.57	0.31	0.06
	MRE (%)	11.3	12.1	15.9
